# Personalized goals of people living with dementia and family carers: A content analysis of goals set within an individually tailored psychosocial intervention trial

**DOI:** 10.1002/trc2.12493

**Published:** 2024-07-15

**Authors:** Jessica Budgett, Andrew Sommerlad, Nuriye Kupeli, Sedigheh Zabihi, Kenneth Rockwood, Claudia Cooper

**Affiliations:** ^1^ Centre for Psychiatry and Mental Health Wolfson Institute of Population Health Queen Mary University of London London UK; ^2^ Division of Psychiatry University College London London UK; ^3^ Islington Memory Service, Camden and Islington NHS Foundation Trust London UK; ^4^ Marie Curie Palliative Care Research Department, Division of Psychiatry London UK; ^5^ Preventive Neurology Unit, Wolfson Institute of Population Health Queen Mary University of London London UK; ^6^ Divisions of Geriatric Medicine & Neurology Department of Medicine Dalhousie University Halifax Nova Scotia Canada; ^7^ Memory Services, East London NHS Foundation Trust London UK

**Keywords:** dementia, Alzheimer's disease, outcome measures, goals, goal attainment scaling, goal setting, individualized care, person centered, psychosocial intervention

## Abstract

**Introduction:**

Person‐centered goals capture individual priorities in personal contexts. Goal Attainment Scaling (GAS) has been used in drug trials involving people living with dementia (PLWD) but GAS has been characterized as difficult to incorporate into trials and clinical practice. We used GAS in a trial of New Interventions for Independence in Dementia Study (NIDUS)‐family, a manualized care and support intervention, as the primary outcome and to tailor the interventions to goals set. We aimed to assess the feasibility and content of baseline goal‐setting.

**Methods:**

We developed training for nonclinical facilitators to set individualized GAS goals remotely with PLWD and family carer dyads, or carers alone, in the intervention trial, during the COVID‐19 pandemic. A qualitative content analysis of the goals set explored participants’ priorities and unmet needs, to consider how existing GAS goal domains might be extended in a psychosocial intervention trial context.

**Results:**

Eleven facilitators were successfully trained to set and score GAS goals. A total of 313/328 (95%) participants were able to collaboratively set three to five goals with the facilitators. Of these, 302 randomized participating dyads set 1043 (mean 3.5, range 3 to 5) goals. We deductively coded 719 (69%) goals into five existing GAS domains (mood, behavior, self‐care, cognition, and instrumental activities of daily living); 324 (31%) goals were inductively coded into four new domains: carer break, carer mood, carer behavior, and carer sleep. The most frequently set goals pertained to social support. There was little variation in types of goals set based on the context of who set them or level of pandemic restrictions in place.

**Discussion:**

It is feasible for people without clinical training to set GAS holistic goals for PLWD and family carers in the community. GAS has potential to facilitate personalization of care and support interventions, such as NIDUS‐family, and facilitate the roll out of more personalized care.

**Highlights:**

- Goal Attainment Scaling (GAS) can capture meaningful priorities of people with dementia and their family carers.
- A psychosocial intervention RCT used GAS as the primary outcome measure and goals were set collaboratively by non‐clinically trained facilitators.
- The findings underscore the feasibility of using GAS as an outcome measure with this population.
- The content analysis findings unveiled the diversity in experiences and priorities of the study participants.
- GAS has the potential to support the implementation of more person‐centred approaches to dementia care.

## 1 INTRODUCTION

An estimated 944,000 UK people live with dementia.^1^ Most want to live at home for as long as possible.^2^ Patient goals and priorities should always guide care,^1^ especially for dementia, where symptoms and their relationship with quality of life are so varied.^3^ Developed in 1968, Goal Attainment Scaling (GAS)^4^ has been modified and applied to areas including cognitive rehabilitation, limb spasticity, and dementia care.^5^, ^6^, ^7^, ^8^ GAS allows participants to set individualized goals with the facilitator that are defined so an independent evaluator can score their attainment. GAS involves identifying problems and priorities, as well as defining, setting, and rating goals—a more complex cognitive process than more standardized outcome measures, leading to concerns it may be impractical in clinical practice,^9^ and may lack methodological specification.^10^, ^11^ Though not tied to one specific methodology, GAS requires formulation of well‐written, specific, measurable, attainable, relevant, and time‐bound (SMART) goals. GAS has been used successfully with people living with dementia (PLWD)^10^, ^12^, ^13^, ^14^, ^15^, ^16^ and their family carers^17^, ^18^, ^19^ to detect small, clinically important intervention effects up to 12 months.^10^ GAS has been used as an outcome in randomized control trials (RCTs) in dementia: mainly drug trials,^13^, ^20^, ^21^ or within an intervention.^19^, ^22^, ^23^ Several studies have analyzed goal content to summarize patient and carer priorities for care and unmet needs.^20^, ^21^, ^24^, ^25^

The New Interventions for Independence in Dementia Study (NIDUS)‐family RCT^26^, ^27^ was the first large trial where GAS goals were the primary outcome and used to tailor an intervention.^26^ The NIDUS‐family study trained nonclinical facilitators, with the intention of developing an intervention that was scalable and affordable.^27^ Participants set goals around what would help the PLWD to live for as long and as well as possible in their own homes. We analyze the content of these goals, set during the pandemic when PLWD and their carers faced service curtailment and severe risks from COVID‐19.^28^ The pandemic provided an opportunity to assess the feasibility of goal setting remotely.

To explore priorities related to living well at home, we aimed to describe (1) the methodology used to enable nonclinical facilitators to set goals; (2) the feasibility of remote GAS goal setting for PLWD and carer dyads with nonclinically trained facilitators; and (3) the goal content. To explore the impact of contexts, we reported how goals varied by (1) who set them (dyad or carer alone and carer relationship), and (2) the pandemic restrictions in place.

## 2 METHODS

### 2.1 Study design

We report baseline findings from the NIDUS‐family multi‐site RCT (ISRC TN11425138),^26^, ^27^ approved by Camden & Kings Cross Research Ethics Committee (19/LO/1667). The trial evaluated NIDUS‐family's manualized, modular care and support intervention, which was tailored to goals participants set plus usual care, versus usual care alone. GAS was the primary trial outcome. The intervention group received six to eight remote sessions (in person if COVID‐19 restrictions allowed) over 6 months, then telephone follow‐ups every 1 to 2 months for up to 1 year.

### 2.2 Study population

From March 2020 to May 2022, we recruited participants from 21 sites across England via professionals working in National Health Service (NHS) trust memory clinics, older adult mental health services, general practitioner practices (in London, Bradford, Leeds, Hull, Oxfordshire, Buckinghamshire, Kent, and Surrey), and via the Join Dementia Research (JDR) database,^29^ carer groups, social media, and local advertisements.

**RESEARCH IN CONTEXT**

1. **Systematic review**: Goal Attainment Scaling (GAS) has been used successfully with people living with dementia or their family carers in previous studies. The New Interventions for Independence in Dementia Study‐family randomized control trial is the first large trial where GAS goals, set by nonclinical facilitators, are the primary outcome evaluating a care and support intervention. The methodology of goal setting and goal content were explored.
2. **Interpretation**: Our findings show it is feasible for people without clinical training to set individualized, holistic goals in this population with appropriate training. Baseline goals were coded within nine overall goal domains relating to living well at home including four new goal domains related to carer well‐being. The most frequently set goals pertained to social support.
3. **Future directions**: GAS can be delivered by nonclinically trained facilitators and has the potential to increase person‐centeredness of trial outcomes for end users of new dementia interventions, including care and support interventions. This can facilitate the roll out of more personalized care.

We recruited people with a documented dementia diagnosis of any type and severity, living in their own homes (including sheltered accommodation), alone or with others. Participants were required to have an English‐speaking family member or friend (henceforth described as “family carer”) in contact at least weekly. We excluded anyone expected to be in the last 6 months of life or enrolled in other interventional research. We excluded dyads where the family carer could not specify at least three goals to support the PLWD living well at home, after a 1‐h goal‐setting session from the trial. We will report the number and percentage of dyads unable to set goals as a measure of GAS feasibility.

### 2.3 Procedures

Study procedures were remote (video call, telephone, and posting study materials) due to the pandemic. Participants gave written, or verbal recorded, informed consent. For PLWD who lacked capacity, their family carer signed a consultee declaration form. Participants completed a baseline assessment (see protocol^26^), of which sociodemographic details are reported here (Table 1). Facilitators were psychology or social science graduates without formal clinical training, who were trained and supervised by a clinical psychologist. They conducted the GAS goal‐setting interview at a second appointment, after completion of baseline measures, with the family carer (and PLWD wherever possible). Before the meeting, they asked participants to identify target areas to improve to enable the PLWD to live as well and for as long as possible at home. Facilitators had an open but focused discussion to explore key issues before setting three to five SMART goals.^30^ In identified areas, co‐facilitators asked participants to detail baseline functioning (recording this current situation as level 0). They then guided formulation of the 5‐point scale (Appendix A), by asking what the baseline situation would look like if much better (+2), a little better (+1), a little worse (−1), and much worse (−2) at follow‐up. Facilitators were encouraged not to exceed 15 min of discussion per goal, and if they did to consult the study team prior to a further appointment. The goals needed to be attainable through the NIDUS‐family intervention, relevant to the dyad's dementia and caring experiences, with at least one goal per dyad focused on the PLWD. Facilitators wrote notes, which they used to draft goals using the goal record sheet (see Appendix A) during or after the appointment. A total of 50 interviews were audio recorded.

**TABLE 1:** Baseline characteristics of people living with dementia (PLWD) and family carers.

	PLWD (*N* = 302)		Family carers (*N* = 302)	
**Age** (years), mean (SD)	79.9	(8.2)	63.4	(12.5)
**Ethnicity**				
White	266	(88.1)	266	(88.1)
Mixed	4	(1.3)	4	(1.3)
Asian	17	(5.6)	16	(5.3)
Black	11	(3.6)	11	(3.6)
Other	4	(1.3)	5	(1.7)
**First language**				
English	260	(86.1)	277	(91.7)
Other	42	(13.9)	25	(8.3)
**Gender**				
Male	133	(44.0)	90	(29.8)
Female	169	(56.0)	212	(70.2)
**Marital status**				
In relationship	178	(58.9)	251	(83.1)
Divorced	17	(5.6)	11	(3.6)
Single	7	(2.3)	35	(11.5)
Widowed	100	(33.1)	5	(1.7)
**Highest educational attainment**				
Higher degree	33	(11.1)	55	(18.2)
Degree	56	(18.9)	97	(32.1)
A level (or equivalent)	58	(19.2)	80	(26.5)
GCSE (or equivalent)	102	(33.8)	55	(18.2)
No formal qualifications	47	(15.9)	15	(5.0)
**Living situation**				
Live alone	84	(27.8)	153	(50.7)
Live with partner/spouse	159	(52.6)	137	(45.4)
Live with children	39	(12.9)	1	(0.3)
Other	20	(6.6)	11	(3.6)
**Cohabiting**				
Dyad are cohabiting	193	(63.9)		
Dyad live apart	109	(36.1)		
**Accommodation**				
Council rented	20	(6.6)	13	(4.3)
Housing association rented	14	(4.6)	217	(71.9)
Private rented	31	(10.3)	12	(4.9)
Owner‐occupied	237	(78.5)	27	(8.9)
**Carer relationship**				
Wife/partner			99	(32.8)
Husband/partner			52	(17.2)
Daughter			109	(36.1)
Son			35	(11.6)
Other			7	(2.3)
**Dementia diagnosis**				
Alzheimer's disease	139	(46.0)		
Vascular dementia	38	(12.6)		
Lewy body dementia	10	(3.3)		
Frontotemporal dementia	8	(2.6)		
Other	84	(27.8)		
Unable to specify	23	(7.6)		

*Note*: Results are *n* (%) unless specified otherwise.

Abbreviations: GCSE, General Certificate of Secondary Education; SD = standard deviation.

Regarding facilitator training and supervision, the training comprised two initial days by an expert team, then cascaded to new starters by J.B./C.C. Facilitators received a further 2‐h training session led by a clinical psychologist, to teach skills in motivational interviewing, opening and closing conversations, managing difficult or upsetting topics, safeguarding procedures, and key considerations of setting and writing SMART goals. This training was manualized to ensure all facilitators received the same content. Following this training, facilitators completed 4 to 8 h of goal‐setting role‐play practice with other facilitators and patient and public involvement members who were current or former family carers. All facilitators completed a final sign‐off with research team leads to check that SMART GAS goals were set efficiently before conducting fieldwork.

Facilitators discussed all goals with C.C. or J.B., who reviewed their SMART criteria based on the fidelity checklist (Appendix B). A GAS expert, K.R., reviewed half of the goals set in the first 3 months. J.B. listened to 10 audio‐recorded GAS sessions (randomly selected across seven facilitators) and completed fidelity checklists (Appendix B) developed by the team, rating five process factors regarding whether goals were set according to the participants’ needs and priorities on a Likert scale from 1 (not at all) to 5 (very much).

Facilitators entered goals into a “goal mapping” spreadsheet where they outlined the 5‐point scale, who set the goal (dyad or carer only; and relationship of the carer to the PLWD), and goal‐setting dates. These were coded based on a published timeline of UK coronavirus lockdown restrictions^31^ into no restrictions (NR), some restrictions (SR), tight restrictions (TR), and lockdown (LD).

### 2.4 Analysis

We describe sample sociodemographic characteristics using standard summary statistics. We conducted content analysis of goals using published methods.^32^, ^33^ Content analysis is a systematic classification process for coding and identifying themes or patterns.^32^

**FIGURE 1:**
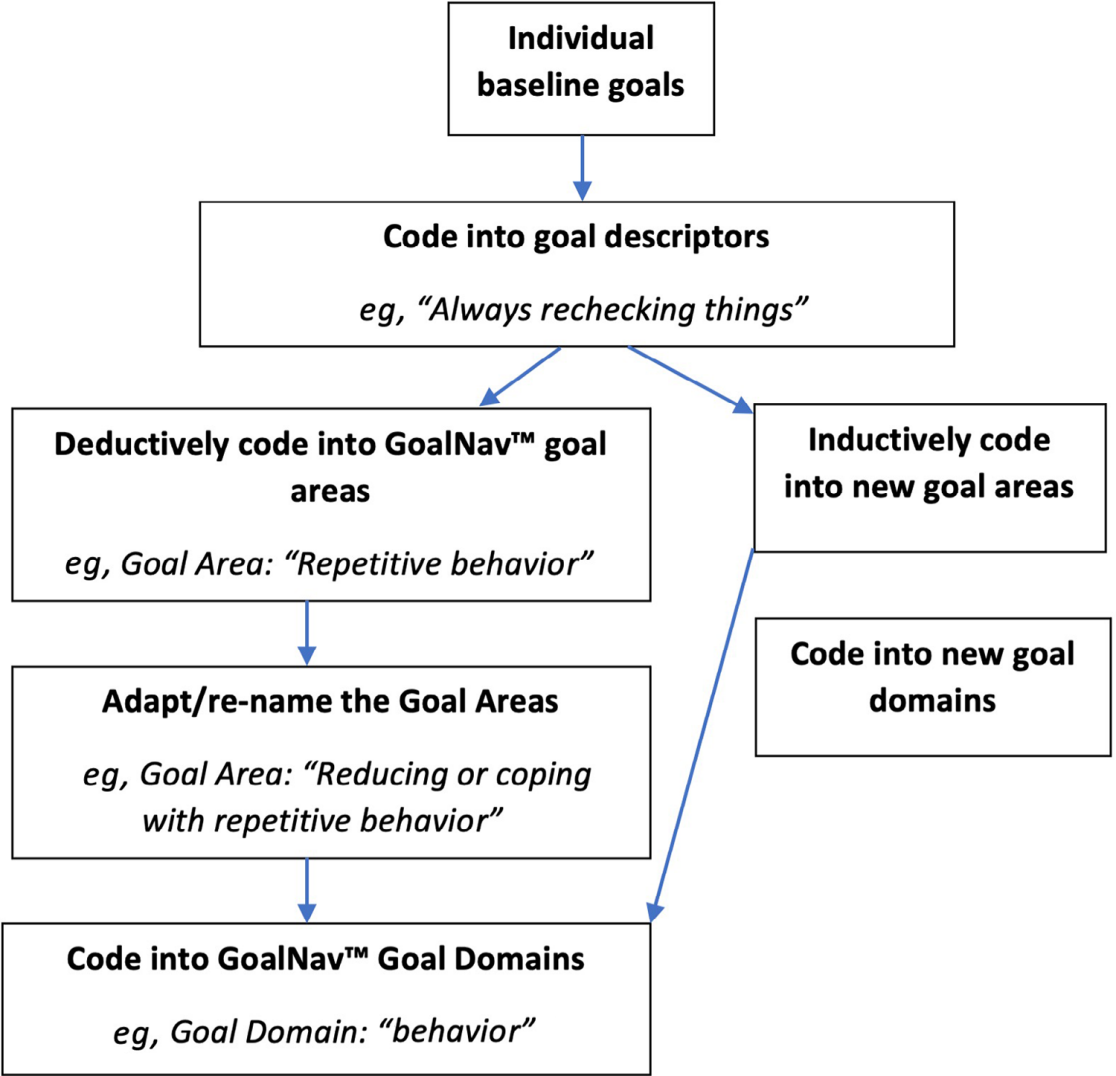
Flow chart showing the content analysis process for coding baseline goals.

Goals in the “goal mapping” spreadsheet were initially deductively coded^32^ using the GoalNav Alzheimer menu (SymptomGuide Dementia), created by K.R. and D.G.I. Clinical Inc (now Ardea Outcomes).^34^, ^35^ This symptom tracking tool provides a library of common and distressing symptoms coded into symptom areas and overall domains, allowing users to identify symptoms of concern and track any changes over time. These symptoms were collated from K.R.’s previous GAS studies and qualitative research exploring individuals’ priorities.^35^ In consultation with K.R., J.B. tailored the GoalNav menu to the NIDUS‐family psychosocial intervention context, for example, adding goals related to carers’ priorities. Goal coding took place throughout the study. Facilitators summarized the central issue as a “goal descriptor.” These were grouped into “goal areas,” then five overall goal domains: behavior, cognition, mood, instrumental activities of daily living (IADLs), and self‐care (Figure 1). Goals that did not align with the existing goal menu were coded as “new goal area.” The coding framework was refined and discussed during team meetings and developed during the content analysis.

Once data collection was complete, J.B. and S.Z. independently coded all goals using the steps above, discussing differences with each other and the initial coding with C.C. to reach consensus. We measured the percentage agreement between their ratings and facilitator ratings. They considered whether goals within the “new goal area” could be coded into the existing GoalNav menu areas and domains, then coded the remaining goals using an inductive content analysis approach.^32^ When goals could be coded into more than one area or domain, the area that best matched the expressed intention of the goal, as outlined in the goal record sheet or goal notes, was selected. For example, most goals related to finding activities that “provide mental stimulation” were set with the expressed intention of improving mood rather than cognition, so were coded in the mood domain.

We report the frequency of goal areas and domains in the final framework, overall and by (1) who set the goal (the dyad together or the carer alone), (2) relationship of the dyad (spouse/partner, child or other), and (3) level of UK government coronavirus restrictions when the goal was set.

## 3 RESULTS

### 3.1 Recruitment and baseline characteristics

A total of 328/502 (65%) eligible dyads referred to the study consented to take part, of whom 26/328 (8%) were not randomized: 15 (5%) due to being unable or unwilling to set three to five goals after a 1‐h GAS goal setting session. Others withdrew before randomization because the care recipient died (*n* = 3), had severe health problems (*n* = 5), or for unspecified reasons (*n* = 3). Table 1 describes characteristics of the 302 participant dyads who set GAS goals and were randomized into the trial.

### 3.2 Goal setting process

Participating dyads set 1043 individual goals (mean 3.5 goals per dyad, ± 0.6; range 3 to 5), with 11 facilitators. Goals were set by the family carer (*n* = 258; 85%) (in two cases with a second family member), or the family carer and care recipient together (*n* = 44; 15%). Involvement of the PLWD was supported where possible, but often the family carer considered that they lacked capacity to understand the process (*n* = 152; 50%), or that it would not be beneficial. Regarding the means of communication, 215 (71%) participants set goals via video call, 81 (27%) via telephone, and 6 (2%) in person.

The goal‐setting session lasted on average 43 (± 8; range 18 to 72) min. Facilitators formulated goals with 104 (34%) dyads after one goal‐setting session; for 180 (60%) dyads, the facilitator additionally conferred with the dyads by a short phone call to clarify minor adjustments advised by the supervisory team to increase goal utility of “SMARTness.” Eighteen dyads (6%) required a second meeting to substantially revise their GAS goals. The GAS expert reviewed 32 goals (50%) of 10 dyads. Feedback suggested ways to adapt goal setting to lockdown restrictions and encouraged the use of participants’ own language (eg, “switch off”) and defining such terms (ie, “a break from thinking about caring responsibilities”) to balance individualization and standardization.

J.B. completed 10 fidelity checklists for four dyads and six carer‐only recorded goal‐setting sessions. All facilitators kept the PLWD central to the goals, set goals according to their priorities outlined by participants, used participants’ language, and kept the carer and PLWD engaged (all rated 5). For the four dyads setting goals, three facilitators managed contrasting or unrealistic expectations of the carer and PLWD very well (rated 5), while one facilitator was rated as 3 for prioritizing the carer's over the PLWD's suggestions for one goal. One facilitator was rated as 2 for not effectively managing unrealistic expectations, and this goal was changed during the review process.

### 3.3 Goal domains and areas (Tables 2 and 3)

A total of 719 (69%) goals focused on the PLWD's needs and priorities. We coded these into the five GoalNav menu domains (mood, behavior, self‐care, cognition, and IADLs). The agreement between J.B. and S.Z.’s coding was 94%, and between their final consensus coding and the facilitators was 90%. The remaining 324 (31%) goals focused on carer well‐being. J.B. and S.Z. inductively coded these into four new domains: carer break, carer mood, carer behavior, and carer sleep.

Table 2 gives examples of how target unmet needs were identified and goals operationalized. Table 3 outlines the goal domains and areas, with examples of the unmet needs addressed in each. Table 4 describes the proportion of goals set in each domain, comparing family carers setting goals alone and with care recipients, carer relationships, and goals set by the level of pandemic restrictions. These factors had little influence on the domains in which goals were set, save for carers alone setting more carer‐focused goals, compared with dyads. Children of PLWD set more goals related to self‐care and spouses more goals to have a caring break; more goals related to PLWD's self‐care were set during lockdown than at other levels of restrictions.

**TABLE 2:** Examples of how Goal Attainment Scaling (GAS) goals were developed by facilitators in goal setting interviews.

Goal domain/goal area	Extracts from recorded GAS setting interviews	Adapted +2 level of GAS goal
Mood/improving interest/initiative	*Husband*: “I think she would like to go back to the theatre and visit some museums … she really enjoys art and culture”	+2: PLWD has been able to enjoy art and culture once a month either in person or online.
Behavior/increasing positive interactions with others	*PLWD*: “I would like my family to visit more … I see them about once a month at the moment … would like to see them once a week”	+2: PLWD has social contact with family or friends (other than primary carer) once a week for at least 15 min (in person or via video call or telephone).
Self‐care/improving personal care	*Partner*: “She is resistant to any kind of help with personal hygiene, it's causing health problems … she will shout or push me.”	+2: PLWD is accepting of help with regards to her personal care, 50 % of the time
Cognition/reducing or coping with repetitive questions/stories	*Daughter*: “I just want him to stop asking the same question about where mum is every day … it is horrible having to explain she has died repeatedly.”	+2: Carer feels distressed in response to PLWD's repetitive questions about her mum once a week or less.
IADL/operating gadgets or appliances	*Daughter*: “He is leaving the iron or oven on, it sometimes sets smoke alarm off … I'm worried about safety, but he loves cooking …”	+2: PLWD leaves the oven, or iron on once a fortnight or less and continues to use the appliances.
Carer break/carer has more time for other activities	*Daughter*: “I would like to do more exercise … I love swimming so really want to go back to the pool when it is open again”	+2: Family carer exercises for one hour a week such as swimming or another enjoyable activity.
Carer mood/reducing carer worry about the future	*Wife*: “I keep getting feelings of panic about what will happen if something happens to me … how will he cope.”	+2: Family carer feels intense worry or panic symptoms about future care of PWLD only occasionally (once a week or less)
Carer behavior/ Improving carer reaction to PLWD	*Son*: “Every day she keeps misplacing and hiding the house key and home phone … I get so frustrated and I end up angry and shouting … .”	+2: Carer is expressing frustration (eg, shouting) towards PLWD when items go missing on 2 days a week or less.
Carer sleep/reducing carer sleep problems	*Husband*: “I get so worried about her (PLWD), it keeps me up at night most nights.”	+2: The carer has intense worries about partner that disturb his sleep once a week or less.

Abbreviations: IADL, instrumental activities of daily living; PLWD, people living with dementia.

**TABLE 3:** Description of unmet needs addressed in goal domains and areas.

Goal domain (*N*, % all goals)	Goal area	Number of goals	Example of unmet need/problem
**Mood 245 (23%)**	Improving interest/initiative	168	PLWD is not engaging in activities that provide mental stimulation resulting in low mood
	Reducing anxiety/worry	45	PLWD is tense and stressed throughout the day
	Reducing low mood	32	PLWD is easily tearful/upset/crying spells
**Behavior 242 (23%)**	Increasing positive interaction with others (including coping with changed relationships)	120	PLWD has limited interaction with others including family and friends.
	Reducing or coping with aggression, irritability, frustration, restlessness, shadowing, repetitive behavior, or unsafe actions	73	PLWD is quick to anger during conversations with carer (“flies off the handle easily”); or PLWD does not like being left alone more than a few minutes
	Improving sleep disturbances	49	PLWD is waking up distressed in the night
**Self‐care 155 (15%)**	Reducing physical complaints	84	PLWD has very limited walking ability
	Improving personal care or dressing	33	PLWD becomes distressed during personal care
	Improving appetite or fluid intake	28	PLWD is eating less, resulting in weight loss
**Cognition 47 (5%)**	Ability to participate in hobbies/ games	21	PLWD is very easily distracted; cannot engage in previously enjoyed activity
	Coping with or improving memory, repetitive questions/stories, or misplacing objects	17	PLWD cannot remember if they have taken their medication or not.
	Improving/coping with concentration or orientation problems	9	PLWD cannot follow conversations or instructions
**IADL/ADL 30 (15%)**	Meal preparation/cooking	13	PLWD is no longer helping prepare meals
	Household chores	12	PLWD is no longer helping with house cleaning
	Operating gadgets/appliances	5	PLWD not able to use new kitchen appliances since moving house
**Carer break 138 (13%)**	Carer has more time for other activities	123	Carer has no time for sociable activities
	Accessing more care support/overnight respite	15	The carer needs more help with caring (family or professional)
**Carer mood 99 (10%)**	Reducing carer worry about the future	47	Carer is worried about the future care of PLWD if something happens to them
	Reducing carer worry anxiety	39	Carer is having feelings of panic when caring
	Reducing carer low mood	13	Carer is easily tearful and upset
**Carer behavior 79 (8%)**	Improving carer reaction to PLWD	57	Carer shows irritability/frustration via shouting in reaction to PLWD behavior
	Carer accessing more resources	22	Carer does not have enough external support and does not know how to access it
**Carer sleep 8 (1%)**	Reducing carer sleep problems due to worry or PWLD's behavior	8	PLWD wakes up carer multiple times during nights

Abbreviations: ADL, activities of daily living; IADL, instrumental activities of daily living, PLWD, people living with dementia.

**TABLE 4:** Number (%) of goals set by dyads or family carers alone, by carer relationship, and by pandemic lockdown stage, within each goal domain.

Goal domain	Goals set by (*n* %)								COVID‐19 restrictions							
	Dyad		Carer only		Spouse		Child/Other		None		Some		Tight		Lockdown	
	*N* = 44		*N* = 258		*N* = 154		*N* = 148		*N* = 664		*N* = 222		*N* = 105		*N* = 94	
**Behavior**	38	**25%**	204	**23%**	118	**22%**	124	**24%**	144	**22%**	52	**23%**	25	**24%**	21	**22%**
**Cognition**	8	**5%**	39	**4%**	23	**4%**	24	**5%**	31	**5%**	12	**5%**	1	**1%**	2	**2%**
**Mood**	38	**25%**	207	**23%**	121	**23%**	124	**24%**	175	**26%**	64	**29%**	26	**25%**	23	**24%**
**IADL**	5	**3%**	25	**3%**	9	**2%**	21	**4%**	21	**3%**	4	**2%**	4	**4%**	1	**1%**
**Self‐care**	24	**16%**	131	**15%**	69	**13%**	86	**17%**	95	**14%**	27	**12%**	15	**14%**	18	**19%**
**Carer mood**	8	**5%**	92	**10%**	54	**10%**	46	**9%**	63	**9%**	23	**10%**	8	**8%**	6	**6%**
**Carer break**	17	**11%**	121	**14%**	86	**16%**	52	**10%**	86	**13%**	19	**9%**	18	**17%**	15	**16%**
**Carer behavior**	13	**9%**	66	**7%**	44	**8%**	35	**7%**	46	**7%**	19	**9%**	7	**7%**	7	**7%**
**Carer sleep**	0	**0%**	8	**1%**	7	**1%**	1	**0%**	3	**0%**	2	**1%**	2	**1%**	1	**1%**

Abbreviation: IADL, instrumental activities of daily living.

### 3.4 Content analysis

#### 3.4.1 Mood (to improve PLWD's mood)

Most goals in this domain are related to improving the PLWD's interest and initiative, and engagement in “new or more enjoyable activities” to improve mood, and “provide mental stimulation.” Other goals focused on reducing the frequency, duration, or severity of anxiety or low mood symptoms. For example, one PLWD and their spouse set their goal as “PLWD can leave the house without anxiety symptoms (breathlessness, sweating or getting tearful) lasting longer than 3 min, twice a week or more.”

#### 3.4.2 Behavior (to increase positive PLWD behaviors)

The most common goal here related to “increasing positive social interaction with others”; often more contact with people “other than the carer.” Sometimes the goal pertained to the dyadic relationship, usually led by the family carer struggling with their changed relationship and role and wanting “better quality” or more “meaningful” social time with the PLWD. Some goals focused on the amount rather than type of social contact, and the interaction was described flexibly as “in person” or “remotely.” For others, the type of social contact was specified. For example, a daughter caring for her mother who was concerned she had no contact with peers, despite enjoying socializing, set a goal around her mother as “participating in a group activity once a week.”

Other goals in this domain related to aggression, irritability, frustration, restlessness, shadowing, repetitive behavior, or unsafe actions: reducing frequency of such behaviors, or family carer distress about it, where carers felt that reducing its frequency was unrealistic.

Goals related to “sleep disturbances” were around difficulties getting to sleep, waking at night, daytime sleepiness, and day/night disorientation. A spouse who was regularly woken by the PLWD getting dressed believing it was morning gave a +2 description: “PLWD no longer waking his wife up during the night by turning on the lights and getting dressed.”

#### 3.4.3 Self‐care (to improve the PLWD's physical health)

Most self‐care goals described managing pain or reduced mobility. A father and son set a goal to “get out of the house at least two times a week,” classified as mobility‐related because the father found getting in and out of the car painful. Other goals related to reducing the PLWD's resistance or distress during personal care. Goals relating to “improving appetite or fluid intake” were to increase oral intake (*n* = 19); restrict diet (*n* = 11) or increase fluid intake (*n* = 6). A daughter concerned about her mother's weight loss set a goal for “at least one balanced meal a day.”

#### 3.4.4 Cognition (to improve PLWD's participation in hobbies/games or coping with cognitive symptoms)

Most commonly (*n* = 21), these goals aspired to help PLWD overcome cognitive challenges that prevented participation in a favorite hobby or game. In classifying the goals, facilitators considered dyads’ formulation of the unmet need—whether it focused on a cognitive or emotional barrier to participation. For example, a spouse dyad recognized that the PLWD needed to adapt his hobbies due to his poor attention span affecting his ability for previously enjoyable word puzzles. They set a goal of “being able to enjoy a hobby for 30 min, 2 to 3 days a week.”

Other goals focused on “improving or coping with” concentration, orientation or memory problems, repetitive questions, or misplacing objects.

#### 3.4.5 IADL/ADL (to improve the PLWD's involvement in household activities)

Goals focused on the PLWD becoming more involved, engaged, confident, or safe in carrying out IADLs. Most specified that the ADL could be supported. A typical example of goals set around meal preparation and cooking is the PLWD “participating in preparing a meal twice a week (with or without support).”

#### 3.4.6 Carer goals (to increase breaks from caring, improve carer mood, sleep, or increase positive carer behaviors)

Most “carer break” goals were focused on the amount of time to self, but others prioritized needs to access more respite and support, to enable time away from caring. A husband experiencing carer stress set a goal for “external support in place 3 days a week for at least 2 h.”

The most common “carer mood” goal was to worry less about the future. Most of this worry (35/47 goals) related to what would happen to the PLWD if the carer was no longer able to look after them. “Carer sleep” goals often intersected with others, for example, where sleep was disturbed by worries, or they were directly disturbed or woken by the PLWD's unmet needs.

Often during GAS interviews, family carers acknowledged that the PLWD's behavior was a dementia symptom and unlikely to change, so they set goals around how they felt and responded to it, which we classified as “carer behavior” goals. Other goals related to carers taking actions to get more support.

## 4 DISCUSSION

GAS was a feasible method of identifying and setting clinically meaningful goals in a psychological intervention trial. It is more time‐consuming than other standardized quality of life measures (the mean time of 43 min to set 3 to 5 fully scaled GAS goals is similar to previous reports^20^, ^36^), but produces a highly personalized and holistic outcome measure, capturing what is relevant, important to, and desired by PLWD and their carers.

While previous studies used clinician or expert‐led GAS,^37^, ^38^ we report that trained and supervised nonclinical facilitators successfully set SMART goals with good fidelity, as did another recent study.^23^ We involved PLWD in goal setting where possible, but most goals were set with family carers alone. This approach enabled us to include people with all stages of dementia. This is the first study to use GAS remotely. Setting goals via video call or telephone may have impacted their quality if trust building was harder. Skills in establishing trust, active listening, and difficult conversations were taught in facilitator training.^39^ The experienced study team checked goals to maintain their quality.

The most frequently set goals pertained to social support—to engage in activities or for carer respite. The NHS long‐term plan,^40^ integrated care boards,^41^ and other current UK policies prioritize personalized, integrated care,^42^ and GAS may be a valuable tool to support it. The goals capture the diversity of experiences of dementia: Not everyone wants the same result, even for the same target behavior or problem.

The distribution of goal domains set by dyads versus family carers alone was similar, but we cannot know how goals family carers set reflected PLWD's priorities. Carers alone set more carer‐focused goals. Carers often report that it is difficult to think of their own needs when taking on a caring role,^43^ so this structure to set goals may be helpful in clinical practice. Spouses of people with dementia set more goals around respite needs. Spouses report having lower social support than adult children of PLWD^44^ and are more likely to be resident carers; resident carers report greater psychological burden than nonresident carers.^45^ Carers supporting parents set more goals related to managing the PLWD's self‐care, probably reflecting the challenges in addressing self‐care needs when not coresident. Further analysis could directly explore how goals varied between carers who lived with, and separately to, the PLWD.

This paper describes goals that were realistically achievable by NIDUS‐family, a psychosocial support intervention. These were different from those set, for example, in drug trials, which usually focus on improving cognition directly. We found consistency between pandemic periods with varying restrictions, demonstrating GAS's flexibility across contexts. Nonetheless, the feasibility of goal setting in other settings, and in other countries, may differ. We manualized the GAS training to maximize reliability but further work to develop standardized training and methodologies for GAS goal setting in this population would support its implementation in dementia care.

## 5 CONCLUSION

Nonclinical facilitators were effectively trained and supported to set GAS goals. This approach to goal setting is potentially scalable within clinical practice, and useful in intervention trials to facilitate person‐centered, holistic dementia care.

## AUTHOR CONTRIBUTIONS

Jessica Budgett led this study, analyzed the data, and drafted the manuscript. Andrew Sommerlad, Nuriye Kupeli, and Kenneth Rockwood participated in the design of this study and contributed to writing the manuscript. Sedigheh Zabihi participated in the data analysis and contributed to writing the manuscript. Claudia Cooper conceived the larger NIDUS‐family study, participated in the design of this study, and contributed to writing the manuscript. All authors reviewed the manuscript.

## CONFLICT OF INTEREST STATEMENT

K.R. is cofounder of Ardea Outcomes (DGI Clinical until 2021), which in the past 3 years has had contracts with pharma and device manufacturers (Danone, Hollister, INmune, Novartis, Takeda) on individualized outcome measurement. In 2020, on behalf of Ardea Outcomes, K.R. attended an advisory board meeting with Nutricia on dementia. The other authors declared no potential conflicts of interest with respect to the research, authorship, and/or publication of this article. All author disclosures are available in the supporting information.

## CONSENT STATEMENT

All human subjects provided written or audio‐recorded informed consent.

## Supporting information

Supporting Information

Supporting Information

Supporting Information

Supporting Information

## References

[1] Prince M , Knapp M , Guerchet M , et al. Dementia UK: Update. Alzheimer's Society; 2014.

[2] Carter D. Fix Dementia Care: Homecare. Alzheimer's Society; 2016. https://www.alzheimers.org.uk/sites/default/files/migrate/downloads/fix_dementia_care_homecare_report.pdf

[3] Cooper C , Mukadam N , Katona C , et al. Systematic review of the effectiveness of non‐pharmacological interventions to improve quality of life of people with dementia. Int Psychogeriatr. 2012;24(6):856‐870. doi:10.1017/S1041610211002614 22244371

[4] Kiresuk TJ , Sherman RE. Goal attainment scaling: a general method for evaluating comprehensive community mental health programs. Community Ment Health J. 1968;4(6):443‐453. doi:10.1007/bf01530764 24185570

[5] Turner‐Stokes L , Baguley IJ , De Graaff S , et al. Goal attainment scaling in the evaluation of treatment of upper limb spasticity with botulinum toxin: a secondary analysis from a double‐blind placebo‐controlled randomized clinical trial. J Rehabil Med. 2010;42(1):81‐89. doi:10.2340/16501977-0474 20111849

[6] Rockwood K , Joyce B , Stolee P. Use of goal attainment scaling in measuring clinically important change in cognitive rehabilitation patients. J Clin Epidemiol. 1997;50(5):581‐588. doi:10.1016/s0895-4356(97)00014-0 9180650

[7] Stolee P , Stadnyk K , Myers AM , Rockwood K. An individualized approach to outcome measurement in geriatric rehabilitation. J Gerontol Biol Sci Med Sci. 1999;54(12):M641‐M647. doi:10.1093/gerona/54.12.m641 10647971

[8] Kudlicka A , Martyr A , Bahar‐Fuchs A , Sabates J , Woods B , Clare L . Cognitive rehabilitation for people with mild to moderate dementia. Cochrane Database Syst Rev. 2023;6(6):CD013388. doi:10.1002/14651858.CD013388.pub2 37389428 PMC10310315

[9] van Blijswijk SC , Gussekloo J , Heijmans FM , Wind AW , den Elzen WP , Blom JW. Goal attainment scaling with older people in general practice: a feasibility study. Int J Nurs Stud Adv. 2021;3:100015. doi:10.1016/j.ijnsa.2020.100015 38746730 PMC11080302

[10] Budgett J Sommerlad, A , Kupeli, N , Zabihi, S , Olsen, A , Cooper, C. Setting individualised goals for people living with dementia and their family carers: a systematic review of goal‐setting outcome measures and their psychometric properties. Dementia. 2024;23(2):312‐340. doi:10.1177/14713012231222309 38105445 PMC10807246

[11] Cheema K , Dunn T , Chapman C , Rockwood K , Howlett SE , Sevinc G. A systematic review of goal attainment scaling implementation practices by caregivers in randomized controlled trials. J Patient‐Rep Outcomes. 2024;8(1):37. doi:10.1186/s41687-024-00716-w 38530578 PMC10965877

[12] Rockwood K , Fay S , Gorman M. The ADAS‐cog and clinically meaningful change in the VISTA clinical trial of galantamine for Alzheimer's disease. Int J Geriatr Psychiatry. 2010;25(2):191‐201. doi:10.1002/gps.2319 19548273

[13] Rockwood K , Fay S , Song X , MacKnight C , Gorman M. Attainment of treatment goals by people with Alzheimer's disease receiving galantamine: a randomized controlled trial. CMAJ. 2006;174(8):1099‐1105. doi:10.1503/cmaj.051432 16554498 PMC1421447

[14] Rockwood K , Graham JE , Fay S. Goal setting and attainment in Alzheimer's disease patients treated with donepezil. J Neurol Neurosurg Psychiatry. 2002;73(5):500‐507. doi:10.1136/jnnp.73.5.500 12397141 PMC1738123

[15] Rockwood K , Howlett S , Stadnyk K , Carver D , Powell C , Stolee P. Responsiveness of goal attainment scaling in a randomized controlled trial of comprehensive geriatric assessment. J Clin Epidemiol. 2003;56(8):736‐743. doi:10.1016/s0895-4356(03)00132-x 12954465

[16] van Seben R , Reichardt L , Smorenburg S , Buurman B. Goal‐setting instruments in geriatric rehabilitation: a systematic review. J Frailty Aging. 2017;6(1):37‐45. doi:10.14283/jfa.2016.103 28244557

[17] Boots LM , de Vugt ME , Smeets CM , Kempen GI , Verhey FR. Implementation of the blended care self‐management program for caregivers of people with early‐stage dementia (partner in balance): process evaluation of a randomized controlled trial. J Med Internet Res. 2017;19(12):e423. doi:10.2196/jmir.7666 29258980 PMC5750419

[18] Berwig M , Dinand C , Becker U , Halek M. Application of Marte Meo® counselling with people with behavioural variant frontotemporal dementia and their primary carers (AMEO‐FTD)—a non‐randomized mixed‐method feasibility study. Pilot Feasibility Stud. 2020;6:32. doi:10.1186/s40814-020-0551-1 32128250 PMC7043032

[19] Wilz G , Schinköthe D , Soellner R. Goal attainment and treatment compliance in a cognitive‐behavioral telephone intervention for family caregivers of persons with dementia. GeroPsych J Gerontopsychology Geriatr Psychiatry. 2011;24(3):115‐125. doi:10.1024/1662-9647/a000043

[20] Rockwood K , Stolee P , Howard K , Mallery L. Use of goal attainment scaling to measure treatment effects in an anti‐dementia drug trial. Neuroepidemiology. 1996;15(6):330‐338. doi:10.1159/000109923 8930946

[21] Leroi I , Atkinson R , Overshott R. Memantine improves goal attainment and reduces caregiver burden in Parkinson's disease with dementia. Int J Geriatr Psychiatry. 2014;29(9):899‐905. doi:10.1002/gps.4077 24510471

[22] Harris N , Boyd H , Evans N , et al. A preliminary evaluation of a client‐centred prompting tool for supporting everyday activities in individuals with mild to moderate levels of cognitive impairment due to dementia. Dementia. 2021;20(3):867‐883. doi:10.1177/1471301220911322 32249596 PMC8044608

[23] Chester H , Beresford R , Clarkson P , et al. The dementia early stage cognitive aids new trial (DESCANT) intervention: a goal attainment scaling approach to promote self‐management. Int J Geriatr Psychiatry. 2021;36(5):784‐793. 10.1002/gps.5479 33271639

[24] Clare L , Linden DE , Woods RT , et al. Goal‐oriented cognitive rehabilitation for people with early‐stage Alzheimer disease: a single‐blind randomized controlled trial of clinical efficacy. Am J Geriatr Psychiatry. 2010;18(10):928‐939. doi:10.1097/JGP.0b013e3181d5792a 20808145

[25] Regan B , Wells Y , Farrow M , O'Halloran P , Workman B. MAXCOG‐maximizing cognition: a randomized controlled trial of the efficacy of goal‐oriented cognitive rehabilitation for people with mild cognitive impairment and early Alzheimer disease. Am J Geriatr Psychiatry. 2017;25(3):258‐269. doi:10.1016/j.jagp.2016.11.008 28034509

[26] Burton A , Rapaport P , Palomo M , et al. Clinical and cost‐effectiveness of a new psychosocial intervention to support independence in dementia (NIDUS‐family) for family carers and people living with dementia in their own homes: a randomised controlled trial. Trials. 2021;22(1):865. doi:10.1186/s13063-021-05851-z 34857029 PMC8637036

[27] Cooper C , Vickerstaff V , Barber J , et al. A psychosocial goal‐setting and manualised support intervention for independence in dementia (NIDUS‐Family) versus goal setting and routine care: a single‐masked, phase 3, superiority, randomised controlled trial. Lancet Healthy Longev. 2024;5(2):e141‐e151. doi:10.1016/S2666-7568(23)00262-3 38310894 PMC10834374

[28] Tuijt R , Frost R , Wilcock J , et al. Life under lockdown and social restrictions—the experiences of people living with dementia and their carers during the COVID‐19 pandemic in England. BMC Geriatr. 2021;21(1):301. doi:10.1186/s12877-021-02257-z 33971847 PMC8107803

[29] National Institute of Health Research (NIHR) . Join Dementia Research (JDR) Database. Published Feb 24 2015. Accessed May 20, 2024. https://www.joindementiaresearch.nihr.ac.uk/

[30] Bovend'Eerdt TJ , Botell RE , Wade DT. Writing SMART rehabilitation goals and achieving goal attainment scaling: a practical guide. Clin Rehabil. 2009;23(4):352‐361. doi:10.1177/0269215508101741 19237435

[31] Institute for Government . Timeline of UK Government Coronavirus Lockdowns and Restrictions. Institute for Government. Published Dec 9 2021. Accessed December 11, 2022. https://www.instituteforgovernment.org.uk/data‐visualisation/timeline‐coronavirus‐lockdowns

[32] Elo S , Kyngäs H. The qualitative content analysis process. J Adv Nurs. 2008;62(1):107‐115. doi:10.1111/j.1365-2648.2007.04569.x 18352969

[33] Elo S , Kääriäinen M , Kanste O , Pölkki T , Utriainen K , Kyngäs H. Qualitative content analysis: a focus on trustworthiness. Sage Open. 2014;4(1):2158244014522633. doi:10.1177/2158244014522633

[34] Reeve E , Molin P , Hui A , Rockwood K. Exploration of verbal repetition in people with dementia using an online symptom‐tracking tool. Int Psychogeriatr. 2017;29(6):959‐966. doi:10.1017/S1041610216002180 28274302 PMC5426314

[35] Rockwood K. An individualized approach to tracking and treating Alzheimer's disease. Clin Pharmacol Ther. 2010;88(4):446‐449. doi:10.1038/clpt.2010.68 20856240

[36] Rockwood K , Fay S , Jarrett P , Asp E. Effect of galantamine on verbal repetition in AD: a secondary analysis of the VISTA trial. Neurology. 2007;68(14):1116‐1121. doi:10.1212/01.wnl.0000258661.61577.b7 17404193

[37] Gordon JE , Powell C , Rockwood K. Goal attainment scaling as a measure of clinically important change in nursing‐home patients. Age Ageing. 1999;28(3):275‐281. doi:10.1093/ageing/28.3.275 10475864

[38] Hartman D , Borrie MJ , Davison E , Stolee P. Use of goal attainment scaling in a dementia special care unit. Am J Alzheimers Dis. 1997;12(3):111‐116. doi:10.1177/153331759701200303

[39] Renouf P , Budgett J , Wyman D , Banks S , Poppe M , Cooper C. Non‐clinically trained facilitators’ experiences of remote psychosocial interventions for older adults with memory loss and their family carers. BJPsych Open. 2023;9(5):e174. doi:10.1192/bjo.2023.558 37791537 PMC10594201

[40] National Health Service (NHS) . The NHS Long Term Plan. Updated August 21 2019. Accessed December 9, 2022. https://www.longtermplan.nhs.uk/

[41] National Health Service (NHS) . *The Integrated Care Boards (Establishment) Order* 2022. NHS England. Published July 1 2022. Accessed June 14, 2023. https://www.england.nhs.uk/publication/integrated‐care‐boards‐in‐england/

[42] Kim SK , Park M. Effectiveness of person‐centered care on people with dementia: a systematic review and meta‐analysis. Clin Interv Aging. 2017;12:381‐397. doi:10.2147/cia.S117637 28255234 PMC5322939

[43] Clemmensen TH , Lauridsen HH , Andersen‐Ranberg K , Kristensen HK. “I know his needs better than my own”—carers support needs when caring for a person with dementia. Scand J Caring Sci. 2021;35(2):586‐599. doi:10.1111/scs.12875 32410395 PMC8246922

[44] Rigby T , Ashwill RT , Johnson DK , Galvin JE. Differences in the experience of caregiving between spouse and adult child caregivers in dementia with lewy bodies. Innov Aging. 2019;3(3):igz027. doi:10.1093/geroni/igz027 31528714 PMC6736163

[45] Brini S , Hodkinson A , Davies A , et al. In‐home dementia caregiving is associated with greater psychological burden and poorer mental health than out‐of‐home caregiving: a cross‐sectional study. Aging Ment Health. 26(4):709‐715. doi:10.1080/13607863.2021.1881758 PMC8959387 33554655

