# Supplementary material for: Personalized goals of people living with dementia and family carers: A content analysis of goals set within an individually tailored psychosocial intervention trial
Source: Alzheimers Dement (N Y). 2024 Jul 15;10(3):e12493. doi: 10.1002/trc2.12493 (PMC11247364; doi:10.1002/trc2.12493)
Supplement: Supplementary file 2 — Supporting Information [file TRC2-10-e12493-s004.docx]

**Appendix A: Example of a GAS goal setting record sheet and set goal**

| **Goal Area:** | **‘Getting out of the house more’** | **6 or 12-month follow up** | |
| --- | --- | --- | --- |
|  |  | Carer Rating | Clinician rating |
| **Much Better**  **(+2)** | The person living with dementia is going to town at least twice per week, with support. |  |  |
| **Goal**  **(+1)** | The person living with dementia is going to town at least once per week, with support. |  |  |
| **Baseline**  **(0)** | The person living with dementia is currently going to town on an ad hoc basis, on average, three times per month, but it is not part of his regular routine. He tends to go to town with the family carer when she is available, as they both agree that he needs to be accompanied and would not feel confident going alone. The person living with dementia said he enjoys town as he likes being ‘out and about’, ‘seeing people’ and going to a coffee shop or the supermarket, and would like to be doing this on a more regular basis. |  |  |
| **Worse**  **(-1)** | The person living with dementia is going to town twice per month, with support. |  |  |
| **Much Worse**  **(-2)** | The person living with dementia is not going to town at all. |  |  |
