# Supplementary material for: Personalized goals of people living with dementia and family carers: A content analysis of goals set within an individually tailored psychosocial intervention trial
Source: Alzheimers Dement (N Y). 2024 Jul 15;10(3):e12493. doi: 10.1002/trc2.12493 (PMC11247364; doi:10.1002/trc2.12493)
Supplement: Supplementary file 3 — Supporting Information [file TRC2-10-e12493-s003.docx]

**Appendix B: Goal setting fidelity checklist.**

**Researcher Name: ____**

**Assessor Name: ____**

**Participant ID: ______________________________**

| Open ended discussion with carer (and PLWD if present) | Tick if done |
| --- | --- |
| Identify at least 3 challenges to independence and discuss how these challenges relate to relative’s dementia. |  |
| Goal 1-5 (score each goal separately) |  |
| 1. **Choose a challenge that is potentially helped by the intervention and meaningful to the carer and the plwd** |  |
| 1. **Creating baseline descriptors** |  |
| Define a single, clear problem |  |
| Use the carers language |  |
| Talk in terms of observable (measurable) change |  |
| Discuss impact on plwd & carer |  |
| Identify duration of the event |  |
| Frequency (optional) how often does the challenges happen (per hour, per day, per week) |  |
| 1. **Creating goals** |  |
| Specific e.g. Goal must only represent one outcome |  |
| Measurable e.g. clear observable attainment criteria, effectively scaled (-2 to +2) |  |
| Achievable e.g. realistic based on current functioning and time scale. |  |
| Relevant to the intervention e.g. Is this an area that the intervention can target |  |
| Relevant to the dyad e.g. reflects something that matters to both. |  |
| Time bound e.g. Sets clear parameters for achievement within timeframe |  |

| Process factors | Please rate facilitator  1 not at all – 5 very much |
| --- | --- |
| Goal set reflects the priorities identified in the open-ended discussion |  |
| Managed (unrealistic or contrasting) expectations of carer (and PLWD if present) |  |
| Kept PLWD central to goals |  |
| Used carers language not technical jargon |  |
| Keeping the carer (and PLWD if present) engaged in the process (avoided going ‘off track’ too much) |  |
| Any additional notes for future GAS training or the impact of COVID-19 or other external factors and how they influenced goals (Free text) | |
|  | |
